# Theoretical-practical evidence in the prevention and promotion of workers’ mental health

**DOI:** 10.47626/1679-4435-2021-746

**Published:** 2021-12-30

**Authors:** Tadeu Lucas de Lavor-Filho, Gabriel Alves Rocha, Eduardo Carvalho de Almeida, Rochelly Rodrigues Holanda, Vilkiane Natercia Malherme Barbosa, Helena Dias Pereira, Thalia Alves Chagas Menezes, Antoniel dos Santos Gomes-Filho

**Affiliations:** 1 Graduate Program in Psychology, Universidade Federal do Ceará (UFC), Fortaleza, CE, Brazil.; 2 Department of Medicine, UFC, Fortaleza, CE, Brazil.; 3 Graduate Program in Linguistics, UFC, Fortaleza, CE, Brazil.; 4 Department of Nursing, UFC, Fortaleza, CE, Brazil.; 5 Department of Psychology, Centro Universitário Vale do Salgado, Icó, CE, Brazil.

**Keywords:** occupational health, mental health, risk factors, health education, saúde do trabalhador, saúde mental, fatores de risco, educação em saúde

## Abstract

Mental health is an important conditioning factor of health in prevention, promotion, and surveillance practices in the intersectoral activity of workers’ health. The goal of this study is to identify, in the scientific literature, risk factors and the promotion of wellbeing, both regarding workers’ mental health. We approached workers’ health in the field of management and research that provide the foundation, through a theoretical-methodological framework, for assistance and intersectoral policies applied to prevention and promotion of workers’ wellbeing. We conducted a systematic review in May 2020 using the Coordenação de Aperfeiçoamento de Pessoal de Nível Superior (CAPES) scientific journal database, with studies published from 2009 to 2019. We followed the Preferred Reporting Items for Systematic Reviews and Meta-Analyses (PRISMA) guidelines for including and excluding variables and performed a fruitful analysis of a final sample of 14 scientific articles. For analyzing our results, we initially described the elements constituting the studies using a form; after this stage, we evidenced 3 axes for discussing the analytical data: a) interconnections of mental health and workers’ health; b) risk factors and workers’ mental health; and c) workers’ mental health education. Studies show the strengthening of workers’ mental health as an element for prevention and promotion in the psychosocial domain, reduction of morbidity and mortality, coping with precarious employment, the assistance and understanding of occupational psychopathologies, and not individualizing occupational illness.

## Resumo

A saúde mental é uma condicionante de saúde importante para as práticas de prevenção, promoção e vigilância na atuação intersetorial em saúde do trabalhador. O objetivo deste artigo é identificar na literatura de artigos científicos os fatores de risco e a promoção do bem-estar, ambos no campo da saúde mental em saúde do trabalhador. Problematizamos a saúde do trabalhador no campo da gestão e de pesquisas que embasam, por meio de aparato teórico-metodológico, políticas assistenciais e intersetoriais aplicadas à prevenção e promoção do bem-estar de trabalhadores e trabalhadoras. Operamos uma revisão sistemática no mês de maio de 2020 no Portal de Periódicos da Coordenação de Aperfeiçoamento de Pessoal de Nível Superior (CAPES), no período de 2009 a 2019. Orientados pelo método Preferred Reporting Items for Systematic Reviews and Meta-Analyses de inclusão e exclusão de variáveis, realizamos uma análise profícua de uma amostra final de 14 artigos incluídos. Na análise dos resultados, inicialmente foi realizada uma descrição de elementos de composição dos estudos através de um formulário-apêndice; após essa etapa, foram evidenciados três eixos de discussão dos dados analíticos: a) Tessituras sobre Saúde Mental e Saúde do Trabalhador; b) Fatores de Risco e Saúde Mental do Trabalhador; e c) Educação em Saúde Mental do Trabalhador. Os estudos evidenciam o fortalecimento de saúde mental do trabalhador como elemento de prevenção e promoção no campo psicossocial, redução da morbimortalidade, enfrentamento da precarização, de assistência e compreensão das psicopatologias do trabalho e não individualização do adoecimento ocupacional.

## INTRODUCTION

Studies on workers’ health have been the object of investigations in various interfaces of health care, focusing on prevention, promotion, and surveillance and aiming primarily at individual and community-level wellbeing. The World Health Organization (WHO) created, in 2007, a Global Plan of Action on Workers’ Health (2008-2014), centered at promoting policies for conceiving and organizing applied health policies, developing epidemiological indicators, creating mechanisms for collecting and studying evidence in this area, and articulating policies and intersectoral pacts in the field of workers’ health.^1^

In Brazil, workers’ health was instituted by the Federal Constitution, Section 200, as an axis of the Unified Health System (SUS).^2^ However, the National Policy for Workers’ Health was created only in 2004 by the Ministry of Health, focusing on reducing accidents, mortality rates, and occupational diseases, in addition to the National Network for Comprehensive Care of Workers’ Health (Rede Nacional de Atenção Integral à Saúde do Trabalhador, RENAST).^3^ In 2005, with Directive No. 1.125 of July 6, 2005, guidelines for increasing information production and strengthening research, formation of human resources, and manager guidance were created. With this guideline, in 2009 RENAST was incorporated to the SUS’ organogram through Referral Centers for Workers’ Health Care (Centros de Referência em Saúde do Trabalhador, CEREST), which act on units specialized in workers’ health. Finally, as a systematic organization of public policy, 2 fundamental decrees were created for its consolidation within the tripartite administration, namely: Decree No. 1.823, of August 23, 2012 — National Policy for Workers’ Health— and Decree No. 7.602, of November 7, 2011 — National Policy on Occupational Safety and Health (Política Nacional de Segurança e Saúde no Trabalho, PNSST).^4,5^

In addition to this scenario of institutional guidelines backed by legal discourse, workers’ health has also advanced by research applied to explaining theoretical models, concrete experiences, and good practices. As an axis for the operation of the SUS, advances in study themes of this area are attributed through the perspective in surveillance in workers’ health grounded on a multidisciplinary and interdisciplinary vision of knowledge on promoting and protecting workers.^6,7^

In this scenario, we prioritize the discussion of impacts that directly permeate workers’ mental health. We agree with Glina et al.,^8^ Guimarães & Grubits,^9^ and Sato & Bernardo^10^ that mental health is an important conditioning factor of health for workers’ health surveillance practices, since studies pointed by these authors reiterate the increasing incidence of occupational diseases with psychopathological presentations. Nevertheless, in addition to identifying variable factors, care should also promote conditions for promoting wellbeing together with environmental, communicational, and psychosocial factors of work.

In this regard, the aim of the present study is to characterize the scientific production of theoretical and/or empirical studies retrieved from the Coordenação de Aperfeiçoamento de Pessoal de Nível Superior (CAPES) scientific journal database in the last 10 years (2009-2019) which approached theoretical-methodological and practical evidence on risk factors and mental health education among workers. We start from the following research questions: 1) “according to the literature, which risk factors damage workers’ mental health?” and 2) “how is health education able to promote wellbeing in workers’ mental health?”

## METHODS

### DATA COLLECTION STRATEGY

We researched for evidence in the scientific literature through a systematic review. Sampaio & Mancini^11^ define a systematic and rigorous technique for bibliographic research, relating evidence from studies in the literature on a defined theme. For this, strategic procedures are required, determined, and systematized for constituting the synthesis of a compilation of causal and interventional relations on a theme. This review method allows the production of new analyses on phenomena and the integration of indicatives that may prompt new diagnoses and guide decisions.

We followed the steps as instructed by Sampaio & Mancini^11^ for performing a systematic literature review, namely: 1) defining a research question that encompasses the problem to be studied; 2) collecting data — studies published and available in databases and/or digital libraries using search strategy tools, such as descriptors and Boolean operators; 3) reviewing and selecting studies — at this stage, we applied inclusion and exclusion criteria to the text elements, such as titles, abstracts and keywords, which should rigorously follow the central theme of the study; 4) analyzing the content of evidence studies in the final data corpus; and 5) synthesizing the results.

On step 1, we chose the following matters as research questions for our study: 1) according to the literature, which risk factors damage workers’ mental health?, and 2) how is health education able to promote wellbeing in workers’ mental health? Through these 2 questions, we sought a complementation between fields, that is, to comprehend evidence that presents risk factors and health care factors in the domain of workers’ mental health surveillance. Correlating different issues is encouraged in a systematic review in order for it to be unprecedented as a synthesis of the theme, according to Donato et al.^12^

On step 2, we searched through the CAPES scientific journal database with a time constraint comprising the last 10 years of publications, defining scientific articles published between 2009 and 2019. We used the Boolean operator AND to retrieve articles from the platform. We used quotation marks between descriptors for recovering compound/whole terms. We chose the CAPES scientific journal database because it reunites various databases. Study retrieval thus tends to reach a higher diversity of articles, spread throughout the following databases: Directory of Open Access Journals (DOAJ); SciELO Colombia; Engineering Research Database; SciELO (CrossRef); Elsevier (CrossRef); SciELO Public Health; MEDLINE/PubMed (NLM); ScienceDirect (Elsevier); PMC (PubMed Central); Scopus (Elsevier); SciELO; Sociological Abstracts; SciELO Brazil; and Social Sciences Citation Index (Web of Science). Table 1 shows the retrieved texts (503). Subsequently, we included only the peer-reviewed studies (421) since they had higher classification levels, and then we excluded repeated texts (38). We reached a final number of 383 articles for reading titles and abstracts according to the Preferred Reporting Items for Systematic Reviews and Meta-Analyses (PRISMA) guidelines for systematic reviews.

**Table 1:** Articles retrieved from the descriptor search

Descriptors and Boolean operator	Articles retrieved	Peer-reviewed	Excluded (repeated)	Corpus for first analysis
*Saúde do Trabalhador AND Saúde Mental*	269	221	-	-
*Saúde do Trabalhador AND Fatores de Risco*	141	115	-	-
*>Saúde do Trabalhador AND Educação em Saúde*	93	85	-	-
Total	503	421	38	383

Source: Coordenação de Aperfeiçoamento de Pessoal de Nível Superior (CAPES) scientific journal database, April 30, 2020.

### ELIGIBILITY CRITERIA

On step 3, we used the PRISMA method for filtering articles considering their titles, abstracts, and keywords. This method consists in choosing 2 investigators for blindly reading the elements of the articles in both classifications with the aim of including studies that comprise the final analysis corpus.^12,13^ We used, as inclusion criteria, the analytical contributions considering risk factors and the promotion of health education in the field of mental health as central and primary elements for discussing the independent study of the article’s theoretical-methodological nature. Articles in foreign languages (English and Spanish) were considered suitable for inclusion in the analysis. Exclusion criteria considered articles where mental health was not the central discussion theme of the studies, knowingly: a) when “mental health” was only mentioned with no exploration or explanation; b) studies presented semiological and psychopathological emphasis; and c) analytical discussion with no definition of “risk factors” or “health education” or “promotion and prevention factors.” After careful reading by the authors of this study, the final corpus containing the articles had only 14 studies for a full analysis of their content and presentation of their evidence results. We observed a variation among these 14 articles regarding year of publication; studies published in 2015 and 2017 were more frequent and those published in 2009 and 2019 were less frequent.

Considering that a systematic review has steps in its construction that require continuation and linearity of the registered syntheses, writing also represents an important function in the presentation and compilation of evidence systematized in the analysis corpus. Therefore, we constructed a form for categorizing article information, and its template is available at the end of this manuscript. We thus restructured our analyses by describing, through examples, the theoretical and methodological claims and research fields of each study, in addition to explaining the results found in studies in order to provide evidence elements for our research question, which encompasses the domains of mental health and workers’ health.

## Results

### DESCRIPTION OF STUDIES

We began the fourth step by tabulating descriptive information of our data. Table 2 shows the 14 articles constituting the final corpus of this systematic review. Initially, we observed that 35.7% of the studies were theoretical (n = 5), whereas 64.3% were empirical (n = 9). Regarding their main language, 13 studies were originally in Portuguese, with Brazilian author affiliations, and only one study was in Spanish, with foreign affiliations. Within our analyses, we also stratified the regional diversity of the authors’ affiliations, and the results were: Brazilian Southeast^14-21^ (n *=* 8), South^22-26^ (n *=* 5), Northeast^27^ (n = 1), North (n = 0), and Center-West (n = 0) regions. We identified the articles by title, authors, journal, and years since publication.

**Table 2:** Database of our systematic review of the literature

Study	Title	Authors	Journal	Year
1	Working conditions and common mental disorder among primary health care workers from Botucatu, São Paulo State	Carvalho & Binder^14^	*Ciência & Saúde Coletiva*	2010
2	“Each Caps is a Caps”: a coanalysis of resources, tools and standards available in the activities of mental health work	Ramminger & Brito^15^	*Psychologia & Sociedade*	2011
3	Work-related illness and health management strategies among community health workers	Camelo et al.^16^	*Revista Enfermagem UERJ*	2012
4	Mental health and quality of life of civil police officers in the metropolitan region of Porto Alegre, Rio Grande do Sul, Brazil	Wagner et al.^22^	*Revista Brasileira de Medicina do Trabalho*	2012
5	The issue of mental health in occupational health surveillance	Leão & Gomez^17^	*Ciência & Saúde Coletiva*	2014
6	Sickness absence due to mental disorders and psychosocial stressors at work	Silva-Júnior & Fischer^18^	*Revista Brasileira de Epidemiologia*	2015
7	Suffering manifestations: dilemmas and challenges for worker´s health surveillance	Leão & Brant^19^	*Physis: Revista de Saúde Coletiva*	2015
8	Work-Related Mental Health: The Challenges for Public Policies	Bernardo et al.^20^	*Universitas Psychologica*	2015
9	Occupational mental health hotline: a discussion on the Brazilian Unified Health Care System (SUS)	Bottega & Merlo^23^	*Revista Polis e Psique*	2016
10	Analysis of occupation health and mental health policies: a proposal of articulation	Perez et al.^24^	*Saúde em Debate*	2017
11	Work clinic in the SUS: possibility of listening to workers	Bottega & Merlo^25^	*Psicologia & Sociedade*	2017
12	Work-related Mental Health Surveillance in Brazil: characteristics, difficulties, and challenges	Araújo et al.^21^	*Ciência & Saúde Coletiva*	2017
13	Mental illness and its relationship with work: a study of workers with mental disorders	Fernandes et al.^27^	*Revista Brasileira de Medicina do Trabalho*	2018
14	Political crossing: organizational culture and moral suffering in public service	Schünke & Giongo^26^	*Revista Psicologia*	2018

On Table 3, following the numbered sequence of presentation of the selected articles, we present their content categories, namely: theoretical contribution, objective, methodological tools, and thematic fields. This last category contemplates the discussion of the theme where evidence of risk factors and health education are articulated with mental health and workers’ health.

**Table 3:** Selected articles and their content specificities

Authors	Theoretical contribution	Objective	Methodological tools	Thematic fields
Carvalho & Binder^14^	Clinic of activity	To explore the relationships between psychological demands, degree of control, and social support at work and the prevalence of CMD in primary health care workers of Botucatu (São Paulo).	Interviews	Primary health care
Ramminger & Brito^15^	Clinic of activity	To increase the dialog between 2 important fields of health care that seem distant from each other, although they approach so many common themes: mental health and workers’ health.	Participant observation; focus group	Specialized care: CAPS
Camelo et al.^16^	Clinic of activity	To identify work-related illness among community health workers and strategies used by these professionals for managing or preventing them.	Bibliographical review	Work relations
Wagner et al.^22^	Workers’ health surveillance	To assess the mental health and quality of life of civil police officers in the metropolitan region of Porto Alegre (Rio Grande do Sul).	Questionnaires: 1) sociodemographic; 2) WHOQOL-BREF; 3) QSG-12	Work relations
Leão & Gomez^17^	Workers’ health surveillance	To contribute to the integration of mental health into workers’ health surveillance, contributing with theoretical subsidies for constructing strategies in this regard within the RENAST.	Document analysis	Work relations
Silva-Júnior & Fischer^18^	Psychodynamics of work	To assess factors associated with work leaves due to mental disorders related to the job, particularly the perception of workers regarding psychosocial aspects of work.	Interview, documents, regulatory policies (document analysis)	Specialized care
Leão & Brant^19^	Workers’ health surveillance	To critically analyze the challenges and dilemmas regarding the integration of mental health within the scope of workers’ health surveillance within the SUS.	Documents, regulatory policies (document analysis)	Specialized care
Bernardo et al.^20^	Theoretical-methodological critical appraisal of work-related mental health	To present a wider perspective to the development of possible public policies aiming for promoting occupational mental health and the prevention of psychic diseases and suffering caused by work activities.	Document analysis	Work relations
Bottega & Merlo^23^	Psychodynamics of work	To discuss the construction of a line of care/listening in workers’ mental health as an expression of the SUS’ work clinic, based on the psychodynamics of work and within the existent network.	Interview, SQR-20 application	Work relations
Perez et al.^24^	Workers’ health surveillance	To analyze legislation by the Ministry of Health, focusing on caring for the worker/user of the SUS, especially considering mental health.	Document analysis	Work relations
Bottega & Merlo^25^	Psychodynamics of work	To subsidize the construction of propositions for an occupational and mental health clinic for the SUS.	Interview, SQR-20 application	Work relations
Araújo et al.^21^	Workers’ health surveillance	To encourage reflections on the current panorama of surveillance actions in this field, their main hindrances, and possibilities of progress.	Document analysis, bibliographical review	Specialized care
Fernandes et al.^27^	Psychodynamics of work	To analyze the perception of patients cared for at a psychiatric hospital in the Northeast region of Brazil on the relationship between their illness and work activity.	Interview	Hospital care
Schünke & Giongo^26^	Clinic of activity	To analyze, through research, the relationship between management practices at public institutions and the mental health of public employees.	Interview	Work relations

CAPS = Center for Psychosocial Care (Centro de Atenção Psicossocial); CMD = common mental disorders; QSG-12 = Brazilian version of the General Health Questionnaire; RENAST = National Network for Comprehensive Care of Workers’ Health (Rede Nacional de Atenção Integral à Saúde do Trabalhador); SQR-20 = Self-Reporting Questionnaire; SUS = Unified Health System; WHOQOL-BREF = The World Health Organization Quality of Life Questionnaire, short form.

On Table 4, we descriptively analyze the percentage of articles per theoretical contribution; 35.7% (n = 5) approached “workers’ health surveillance”.^18,19,21,22,24^ On the other hand, the less frequently observed theoretical contribution was “theoretical-methodological critical appraisal of work-related mental health”,^20^ with only 7.1% of prevalence (n = 1); this was the only foreign study included in this review.

**Table 4:** Theoretical contributions of the studied articles

Category	Sample (n)	Percentage (%)
Workers’ health surveillance	5	42.3
Clinic of activity	4	28.6
Psychodynamics of work	4	28.6
Theoretical-methodological critical appraisal of work-related mental health	1	7.1

Table 5 shows a descriptive analysis of the percentage of methodological tools used in the investigation of research on occupational mental health. The result showed a bigger emphasis on the use of interviews,^14,18,23,25-27^ all of which were semi-structured, with 42.9% (n *=* 6) of the studies, and document analysis of directives, legislation, medical records, and social security data,^16,17,19,20,21,24^ with 35.7% of the studies (n *=* 5). The least frequently used methods were participant observation and focus group,^15^ both in 7.1 % (n *=* 1).

**Table 5:** Methodological tools in the studied articles

Category	Sample (n)	Percentage (%)
Interview	6	42.9
Document analysis	5	35.7
Participant observation	1	7.1
Focus group	1	7.1
Questionnaire/test	3	21.4
Systematic/bibliographical review	2	14.3

The data described by these tables were the first extract for analyzing evidence studies on the structure of research aimed at our topic in the domain of mental health and workers’ health. Still according to Sampaio & Mancini^11^ and Costa & Zoltowski,^13^ we synthesized our results in the following section. We have divided the data extracted from discursive and pragmatic evidence of research narrated by the reviewed articles into 3 theme blocks.

## DISCUSSION

### INTERCONNECTIONS OF MENTAL HEALTH AND WORKERS’ HEALTH

The intersection between mental health and workers’ health is considered, in some of the analyzed studies, in view of the workers’ health surveillance approach, which was used by 35.7% (n *=* 5) of the retrieved studies, as shown in the previous section. Leão & Brant^19^ state that the mission of workers’ health surveillance is to identify and intervene with determinants and conditioning factors of the mental health of workers”^20^ (p. 1272). Workers’ health surveillance is defined by these authors as a collection of actions that influence the suffering of workers at distinct levels and as a key component of the National Health Surveillance System (Sistema Nacional de Vigilância em Saúde, SNVS), which is responsible for an articulate group of actions of health promotion and morbidity and mortality reduction among the worker population by Araújo et al.^21^

These actions should be present from the care of individual cases of more severe suffering and interventions in work organization in order to integrate psychosocial aspects to occupational hazards, to approximations and articulations with income-generating organizations and the insertion of suffering individuals in the job market, for example.^19^ It is thus clear that the scope of workers’ health surveillance should be broad. In addition to its broadness, it should also have an integrative approach, according to Leão & Gomez,^18^ in the sense that one should not consider objective and subjective aspects separately: in summary, the material elements of the work context — work organization, the cultural context, interpersonal relationships — and individual clinical assessment aspects should act together.

Although these aspects are deemed essential, Bernardo et al.^20^ inquire whether these actions aim to comprehend the complexity of the problem or if they only consist in palliative propositions for reducing the loss of productivity in work processes and adapting workers to the prevailing organizational models and conditions. The authors start from a theoretical-methodological critical perspective of work-related mental health to point out that institutional guidelines do not comprehend workers as active individuals in the process of identifying psychosocial risk factors, but as objects of analysis or intervention.

In this sense, work organization exerts a specific effect over workers, and in certain occupational conditions the employer may cause suffering when the worker sees his or her individual history, projects, and hopes come into shock with an organization that ignores them.^16^ This is why Bernardo et al.^20^ highlight the need for contemplating the micro and macro aspects of workers’ mental health, especially considering the sociohistorical conditions of capitalism that determine work characteristics.

Capitalism conditions, social aspects, and work specificities should be intrinsic to an analysis of workers’ health surveillance. This is perceived, for example, when examining the peculiarities of some employment categories. The mental health of health care workers has been the object of study,^16,23^ highlighting the fact that the proximity with other people’s illness experienced by workers of the SUS may make them sick; above all, studies have indicated that the work conditions or situations faced by health care workers have led them to deny the suffering inherent to this activity or even to quit the profession altogether, such is the physical and psychic distress endured by them. Professionals such as civilian police officers also present a high degree of deterioration of their quality of life and mental health due to contact with violence, low salaries, and social devaluation of their professional category; these professionals may reach mental health indices that are similar to those of victims of great catastrophes or individuals with panic disorders.^22^

According to Fernandes et al.,^27^ research on the health-disease-work relation is mainly structured by the framework of occupational psychopathology, allowing an analysis of the psychodynamic of production processes mobilized by the confrontation of an individual against work, where psychic suffering and pleasure are involved. On the other hand, the characterization of a link between clinical presentation and work is one of the difficulties found when investigating and following workers in mental suffering, since there is no consensus for classifying psychic disorders related to work.^25^

One of the analyzed studies declares that this lack of objective criteria that can connect occupational exposure to psychosocial stressors with illness results in the underreporting of work-related diseases, limiting the knowledge of the problem’s dimension. This hampers the formulation of policies for the promotion and protection of workers’ health.^18^

Aiming to comprehend psychic stressors within the work environment, Bottega & Merlo^25^ bring forth the close relationship between changes in new management models (based on individualization, demands, and objectives) with the emergence of malaise and depression at work. Within the same perspective, Perez & Merlo^24^ state that the current management models aim to capture the workers’ subjectivity, resulting in the formation of an identity based on what one does for a living; when work defines who one is, health may be affected.

Bottega & Merlo^25^ discuss that clinical listening at the work environment has positive effects, such as “removing the individual from solitude, from the individualization of his or her suffering and illness, even though it is unique” (p. 8). Listening cares about the suffering being and about actions, most of which are collective, for overcoming and modifying damaging situations that stem from work organization. This thought is in agreement with Schünke & Giongo,^26^ who argue that organizational communication allows interactions, learning, and exchanges within the work environment.

### RISK FACTORS AND WORKERS’ MENTAL HEALTH

Psychosocial disorders are among the main causes of absenteeism at work, being currently identified as the third main reason for the concession of social security benefits in Brazil. However, among the main problems in this discussion lies the difficulty in identifying a causal nexus between psychic illness and occupational stressors as risk factors to workers’ health.^19^ Overall, the selected literature highlights that, regardless of the psychosocial aspect, mental illness presents itself as an increase in physiological and cognitive stress levels, indicating work-related suffering that appears in various forms such as generalized pain, depressive episodes, or even suicide attempts.^25^

In face of this reality and with the growth of neoliberal ideas in business management, a managerialist logic has taken effect within organizations, which may sometimes lead to the appearance of yet unknown psychosocial risk factors^26^ (p. 450). The new management models and ensuing transformations in the manner of operating work (social interaction contexts, economic crises) showed a close relationship with the increase in psychological violence and moral harassment within organizations.^25^

Leão & Brant^20^ emphasize that clinical diagnoses and the establishment of a causal nexus are not devoid of personal interferences when it comes to the moral and ideological values of the health care professional responsible for this assessment. Therefore, we may infer that the assessment of psychosocial risk factors in a work environment both has a necessary relevance to its consideration and presents conceptual and methodological difficulties in its recognition and (under)dimensioning.

Araújo et al.,^21^ on the other hand, state that, when considering mental health, important gaps interfere with dimensioning the problem, since underreporting is still one of the main obstacles; the identification and action over factors that produce these events are also hindered. In this perspective, it is not easy to establish a relationship between illness and workers’ health, as in addition to these factors hampering problem recognition, psychic illness involves many dimensions of the individual and his or her particularities.^27^

Furthermore, when considering social relations, Fernandes et al.^27^ describe that interpersonal relationships influence workers’ mental health. The lack of understanding and outreach by coworkers, as well as the negative environment and emotional overload, indicate that an absence of social support corroborates the perception of workers regarding stressors in the work environment.

When taking into consideration the difference between genders, some results by studies that analyzed the mental health of health professionals showed moderate and high levels of burn-out and common mental disorders (CMD), which showed a higher prevalence among women.^16^ Taking the center stage among psychosocial determinants in this context, the sexual division of labor, unequal gender relations, harassment, and lack of social recognition are considered conditioning factors of psychic suffering and burn-out.^21^

It is important to note that work by itself, as an occupation, interferes with the individual’s psychic wellbeing, with a fundamental role in human life and also leading to psychic suffering processes. Fernandes et al.^27^ emphasize that some demands of the workplace may influence workers’ health just as psychosocial factors. They comprise psychic loads, which are grouped as psychic overload and underload. The former considers situations of prolonged tension, whereas the latter refers to the impossibility of developing one’s mental capacity, the lack of control over work, distancing between groups of subordinates and managers, social isolation in the work environment, role and interpersonal conflicts, and lack of social support.

Moreover, Araújo et al.^21^ state that work structures relationships and singularities, being a path for the construction of one’s identity, satisfaction, and pleasure; it can, in certain conditions, constitute a pathogenic element, thus containing identification relationships as it engraves one’s image and that of the world, which are internalized as belonging to the individual.

Different government entities and initiatives have indicated paths for identifying psychosocial risk factors and proposed actions for facing them.^20^ In Brazil, the National Policy for Workers’ Health is an example of legislation that apparently seeks to treat the problem within the scope of surveillance, aiming to promote and protect workers’ health, as well as to reduce morbidity and mortality due to work.^2^ However, among the main criticisms directed at analyses and interventions focused on psychosocial risk factors, lies the fact that these are deeply connected to employers’ interests.^20^

### WORKERS’ MENTAL HEALTH EDUCATION

Although it is common knowledge that work directly or indirectly affects workers’ health, more organized guidelines and strategies for prevention and promotion of the health of these individuals within the SUS only began to be effectively articulated in 2002, with RENAST.^2^ From possible repercussions of the recognition of the relevance of this theme in the SUS and of devices such as CEREST, an articulation towards promotion, prevention, assistance, surveillance, and rehabilitation of workers is enabled (Directive No. 1.823/2012, p. 1).^2^

Barbosa et al.^28^ have showed that health care work, especially its precarious conditions, demands, and low effectiveness in solving cases, has made the day-to-day of these professionals even more sickening, especially considering mental health. Therefore, one of the relevant prerogatives for the effectiveness and efficacy of workers’ health care strategies is, in general, the implantation of a health education process among workers so that they can be active agents in the prevention and promotion of mental health care in the workplace.

This way, a need emerges for counterposing work organization as it currently stands, since this environment has contributed to worker illness caused by their own system, which produces more competitivity, individualization of performance, disproportionate objectives, among other strategies for increasing productivity that have resulted in workers’ mental illness.^23^

Therefore, among the attributions and competences of workers at the SUS, the need to foster the possibility of dialoguing with this new care perspective also emerges. This brings about a double challenge, since while these professionals are responsible for providing care, they can also become targets as they are also workers that become sick within these care processes.^4^

For Barbosa et al.,^28^ permanent health education is a process that can transform the reality of work, because its actions and strategies emerge from the workers’ day-to-day; it is from this point that movement towards change is thought out, allowing joint actions stemming from what is experienced, producing know-how in health that triggers care processes that are more creative, collective, and lead to improvement and meet the reality of work.

Mental health education process may thus provide workers with new tools and care strategies that dialog with their day-to-day work and that envision their suffering not as an individual cause, but as a part of the work organization itself.^23^ In this sense, a strategy for facing these sickening work conditions is a process of change of this organization that fosters new, less sickening possibilities and that includes workers as active agents in the production of more collective and humanitarian work.^25^

## Final considerations

In conclusion, it is necessary to stimulate research on the collection of panoramic data on mental health within occupational health studies and, above all, within the comprehension of risk factors identified as variables that aggravate illness and make day-to-day work unproductive. In parallel, research efforts towards investigating health education as an axis for guiding and supporting mental health prevention and promotion practices for workers should also be strengthened.

Through the analyzed articles, this study was able to map a scenario of plural investigation practices, whose construction happens through theoretical and practical paths and where the concept of mental health takes on an important emphasis on workers’ health, which ultimately aims to promote wellbeing and quality of life. Workers’ mental health has incorporated the dimensions of analysis, intervention, and fostering of a broader comprehension of health, whose incidence resonates on the integration of psychosocial aspects, reduction of morbidity and mortality. The inclusion of addressing sociohistorical issues of precarious employment, and assistance and comprehension of occupational psychopathologies that do not individualize occupational illness are also noted.

This study also allowed the exploration of risk factor variables approached in different investigations and the recognition of an occupational illness component. This mapping allowed the recognition that stressor variables are present as physical, cognitive, and interpersonal exertion and psychosocial impacts. Considering this last aspect, we emphasize that work-related mental illness reverberates in face of the vulnerability of the work to which one is subjected and who many times does not even reach underreporting. Our investigation also had as key element the capitalist system as a modus operandi that, throughout history, has not put workers’ health first, and as a symbol of this perverse capital investment, mental health was treated as a causal relationship of psychologizing individualized diagnoses, that is, the problem of illness as a subjective cause instead of recognizing social health care.

The emergence of a workers’ health axis in the advancement of social health care committed to a holistic relationship with the health-disease process contributed effectively to the workers’ health field. Studies have referenced the paradigm of health education within the SUS as an example of this way of preventing and promoting health through a broader and political perspective of mental health care as a constant in the workplace and with a significant role in quality of life and work. Health education has been shown to be a continuous strategy for mental health care both in strengthening bonds and subjective wellbeing and in facing psychic diseases and technically supporting public policies.
